# P-1689. Impact of Antibiotic Usage on Antimicrobial Resistance Rates among Gram-Negatives at a COVID-Care Facility

**DOI:** 10.1093/ofid/ofae631.1855

**Published:** 2025-01-29

**Authors:** Gloria Mayela Aguirre-García, Victor Baylon-Valdez, Luisa F Gonzalez-Gonzalez, Rodolfo Medina-Hernandez, Claudia E Guajardo-Lara, Michel F Martínez-Reséndez, Infectious Disease, Mary C Aleman-Bocanegra

**Affiliations:** TecSalud, Monterrey, Nuevo Leon, Mexico; Instituto Tecnológico y de Estudios Superiores de Monterrey, School of Medicine and Health Sciences, Monterrey, Nuevo Leon, Mexico, Monterrey, Nuevo Leon, Mexico; Instituto Tecnológico y de Estudios Superiores de Monterrey, School of Medicine and Health Sciences, Monterrey, Nuevo Leon, Mexico; Instituto Tecnológico y de Estudios Superiores de Monterrey, School of Medicine and Health Sciences, Monterrey, Nuevo Leon, Mexico; Instituto Tecnológico y de Estudios Superiores de Monterrey, School of Medicine and Health Sciences, Monterrey, Nuevo Leon, Mexico, Monterrey, Nuevo Leon, Mexico; Instituto Tecnológico y de Estudios Superiores de Monterrey, School of Medicine and Health Sciences, Monterrey, Nuevo Leon, Mexico, Monterrey, Nuevo Leon, Mexico; Instituto Tecnológico y de Estudios Superiores de Monterrey, School of Medicine and Health Sciences, Monterrey, Nuevo Leon, Mexico, Monterrey, Nuevo Leon, Mexico

## Abstract

**Background:**

Antimicrobial resistance is directly associated with antimicrobial use and abuse, becoming an important public health problem in modern practice. Unchecked prescriptions, noncompliance with antimicrobial stewardship programs, pandemics, and outbreaks contribute to this issue. This study aims to analyze the antimicrobial resistance patterns over the years in a tertiary-level facility compared to antibiotic usage.

E. coli antimicrobial resistance rates and antibiotic usage

**Figure:**
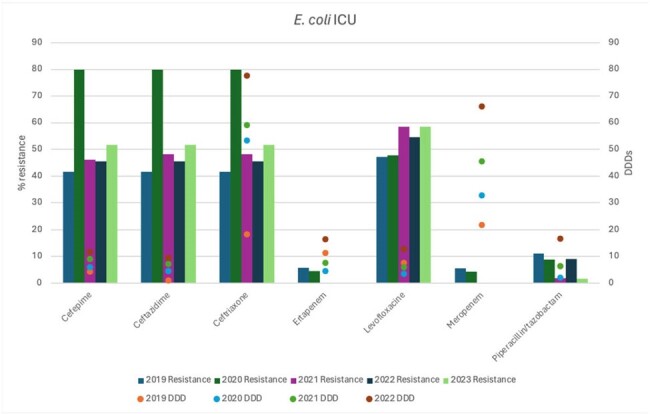

**Methods:**

A retrospective single-center study from 2019 to 2022 was conducted at the Intensive Care Unit of Hospital San Jose, Monterrey, Mexico. Antibiotic usage was calculated using a defined daily dose (DDD) in g/100 beds for Cefepime, Ceftazidime, Ceftriaxone, Ertapenem, Levofloxacin, Meropenem, and Piperacillin/tazobactam. Antimicrobial resistance rates to these antibiotics by *Escherichia coli*, *Klebsiella pneumoniae*, and *Pseudomonas aeruginosa* isolates from ICU were calculated and compared against antibiotic usage over the years.

P. aeruginosa antimicrobial resistance rates and antibiotic usage

**Figure:**
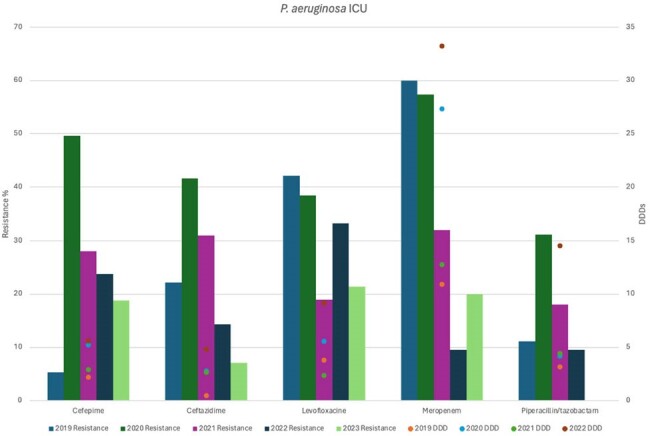

**Results:**

During COVID-19 pandemic in 2020, there was an increased resistance rate of *E. coli* to cephalosporins (ceftriaxone 80%, ceftazidime 80% and cefepime 80%) in comparison to 2019 and the following years. *Klebsiella pneumoniae* had a similar behavior, with high resistance rates to cephalosporins (60% each). *Pseudomonas aeruginosa* isolates also presented a higher resistance rate in 2020 to cefepime (49.6%), ceftazidime (41.6%), meropenem(57.4%) and piperacillin-tazobactam(31.2%) during 2020. During the same year, high DDDs were observed for ceftriaxone (53.3) and meropenem (32.79). Nonetheless, resistance rates to meropenem by *P. aeruginosa* in 2019 and 2020 (60, 57.4%), *E. coli* (5.6, 4.4%) and *K. pneumoniae* (26.3, 7.4%) were not influenced by overuse of meropenem, neither during COVID-19 or the following years.

K. pneumoniae antimicrobial resistance rates and antibiotic usage

**Figure:**
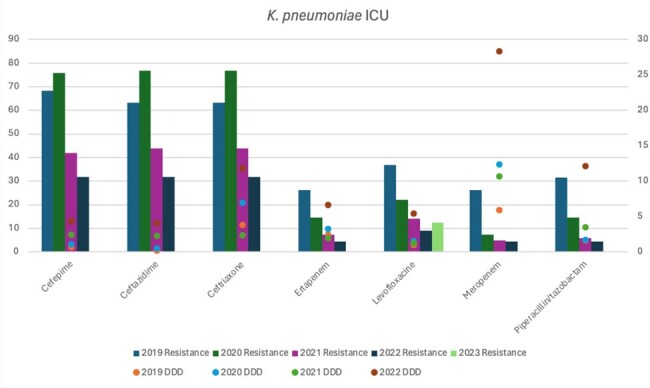

**Conclusion:**

Besides overuse of Beta-lactam antibiotics such as ceftriaxone and meropenem during the COVID-19 pandemic, surpassing importantly recommended DDDs by the WHO, resistance rates to these antibiotics in the following years was not affected. Multidisciplinary teams including an infectious disease specialist were fundamental for antimicrobial stewardship during the pandemic, positively impacting resistance rates of Gram-negatives.

**Disclosures:**

**All Authors**: No reported disclosures

